# Identification of novel homozygous nonsense *SLC10A7* variant causing short stature, amelogenesis imperfecta, and skeletal dysplasia with scoliosis and surgical management of spine

**DOI:** 10.1186/s13023-023-02975-0

**Published:** 2023-11-30

**Authors:** Wenyan Zhang, Ziming Yao, Ruolan Guo, Jun Cao, Wei Li, Chanjuan Hao, Xuejun Zhang

**Affiliations:** 1 Beijing Key Laboratory for Genetics of Birth Defects, MOE Key Laboratory of Major Diseases in Children, Beijing Children’s Hospital, Beijing Pediatric Research Institute, Capital Medical University, National Center for Children’s Health, Beijing, China; 2 Department of Orthopedics, Beijing Children’s Hospital, Capital Medical University, National Center for Children’s Health, Beijing, China; 3 Henan Key Laboratory of Pediatric Inherited & Metabolic Diseases, Henan Children’s Hospital, Zhengzhou Hospital of Beijing Children’s Hospital, Zhengzhou, China

**Keywords:** Next generation sequencing (NGS), Amelogenesis imperfecta, Rare disease, Skeletal dysplasia, Surgical prognosis

## Abstract

**Background:**

Short stature, amelogenesis imperfecta, and skeletal dysplasia with scoliosis is a rare, autosomal recessive, skeletal disorder first described in 2018. This syndrome starts with pre- and postnatal developmental delay, and gradually presents with variable facial dysmorphisms, a short stature, amelogenesis imperfecta, and progressive skeletal dysplasia affecting the limbs, joints, hands, feet, and spine.

**Case presentation:**

We identified a homozygous novel nonsense mutation in exon 1 of *SLC10A7* (NM_001300842.2: c.100G > T / p.Gly34*) segregating with the typical disease phenotype in a Han Chinese family. We reviewed the 12-year surgical treatment history with seven interventions on spine.

**Conclusion:**

To date, only 12 cases of the *SLC10A7* mutation have been reported, mainly from consanguineous families. Our patient showed a relatively severe and broad clinical phenotype compared with previously reported cases. In this patient, annual check-ups and timely surgeries led to a good outcome.

**Supplementary Information:**

The online version contains supplementary material available at 10.1186/s13023-023-02975-0.

## Background

Short Stature, Amelogenesis imperfecta, and Skeletal dysplasia with Scoliosis (SSASKS, OMIM #618,363) was first related to mutations in the solute carrier family 10 member 7 (*SLC10A7*) gene (OMIM #611,459) in 2018 [1]. *SLC10A7* is located on the long arm of chromosome 4 and encodes member 7 of solute carrier family 10, which is a 10-span transmembrane (TM) protein. Patients with SSASKS are characterized by disproportionate short stature, defects of tooth enamel formation, severe scoliosis caused by skeletal dysplasia, facial dysmorphism, hearing impairment, and mildly impaired intellectual development. We report here the first case of SSASKS in a Chinese individual of relatively severe clinical phenotypes and review her 12-year surgical prognosis.

## Case presentation

### Clinical information

A Chinese girl presented at the Orthopedics Department of Beijing Children’s Hospital at 3 years old with progressive scoliosis for surgical treatments. Her mother did not have regular check-ups during pregnancy. She was born full term with an uneventful delivery. Her parents claimed to have a non-consanguineous marriage.

**Fig. 1:**
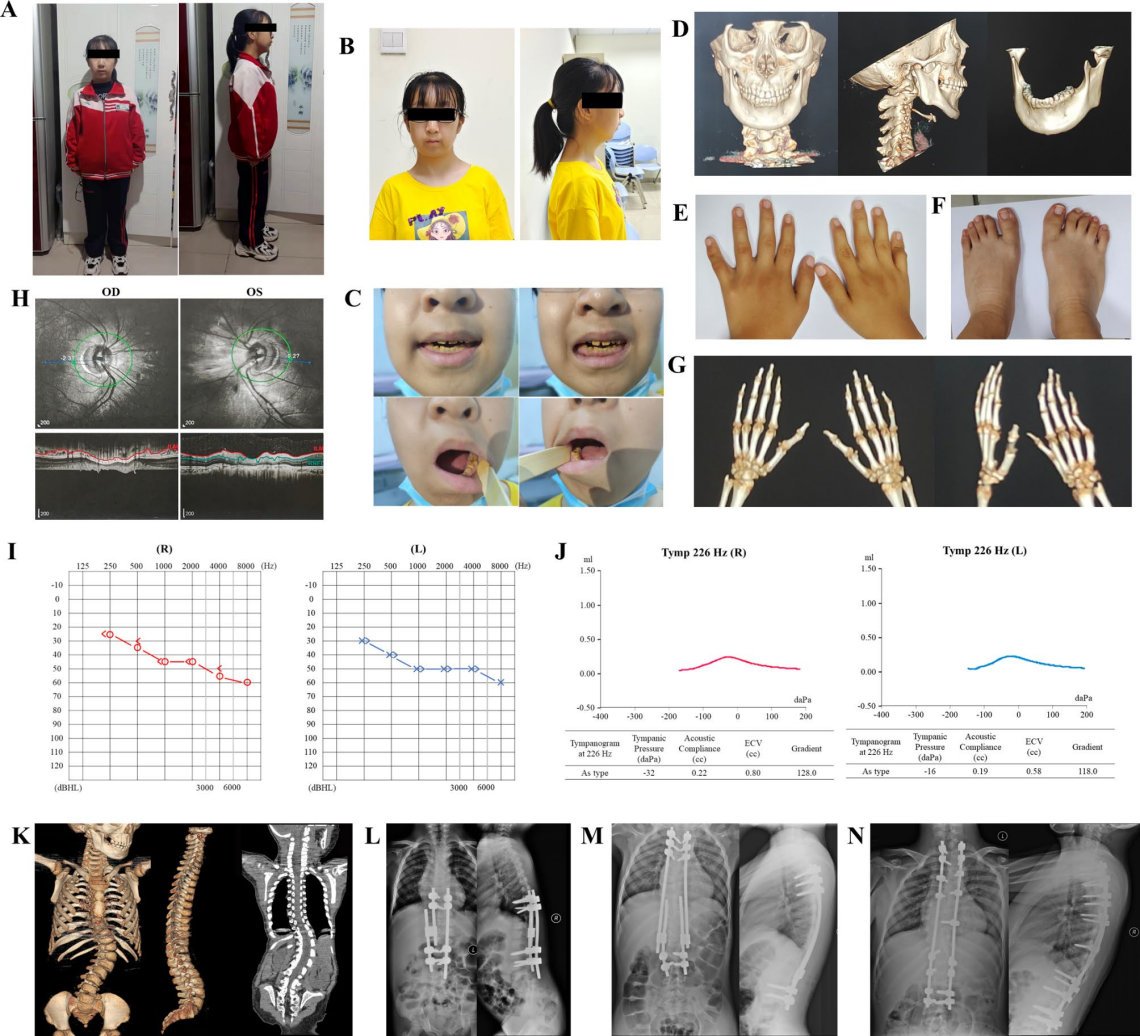
Clinical features of patient. (**A**) Short stature. **(B)** Facial dysmorphism including flat face. **(C)** Hypomineralised amelogenesis imperfecta. **(D)** CT of skull showing flat face and amelogenesis imperfecta. **(E)** Brachydactyly of patient’s hands. **(F)** Mild flat feet. **(G)** CT of hands showed brachydactyly and contracture of the little finger joint. **(H)** Optical Coherence Tomography of patient showed light optic disc color in both eyes, increased the cup-to-disc ratio, optic nerve atrophy and thin optic nerve atrophy. **(I)** Moderate hearing impairment. **(J)** As type tympanogram of patient at 226 Hz of both ears. **(K)** CT images of patient before surgery when 3 years old. Obvious scoliosis was observed at thoracic and lumbar vertebrae. **(L)** The X-ray images of patient after her first single-side growth rod surgery. Pedicle screws were fixed at T9, T10, L3 and L4. **(M)** The X-ray images of patient after surgical replacement of the single-side growth rod by dual growth rod at the age of 10.2 years. Screws were fixed at T1, T2, T3, T5, T8, T11, T12, L2 and L3 at the left side and T1, T2, T4, T5, T10, T12, L2 and L3 at the right side. **(N)** X-ray images of patient after her final fusion surgery. Old screws were removed and a new set of screws was fixed at previous vertebral bodies. Abbreviations, R, right; L, left; OD, Oculus Dexter; OS, Oculus Sinister; ILM, internal limiting membrane; RNFL, retinal nerve fiber layer. T, thoracic; L, lumbar

The girl had a short stature, and by the age of 14 years, her height was 125 cm (< third percentile) and she weighed 32 kg (< third percentile) (Fig. 1A). She had normal neurodevelopment and normal brain density. She had facial dysmorphism, including a flat face, ptosis, retrognathia (Fig. 1B) and type III hypocalcified amelogenesis imperfecta [2] (Fig. 1C, D). Both of her hands and feet had brachydactyly (Fig. 1E, F), and the little finger of the right hand had displayed joint contracture since 10 years old (Fig. 1G). She had moderate hearing impairment in both of her ears. An As-type tympanogram at 226 Hz indicated ossicular fixation (Fig. 1I, J). She had hyperopia and astigmatism since a young age. Optical coherence tomography of the optic disc showed a light optic disc color in both eyes and an elevated cup-to-disc ratio, which indicated optic nerve atrophy. The optic nerve fiber layer was thin (Fig. 1H) and the macula was normal (Supplementary Fig. 1). Radiography was performed at 3, 4, 5, 10, and 14 years old before surgeries. The first time she had radiography performed, a fuzzy atlantoaxial articular surface and irregular vertebral bodies of the thoracic spine were observed (Fig. 1K). Spinal scoliosis developed over time and kyphosis appeared. In addition, she also had decreased hearing, mild hypermetropia, and astigmatism. Echocardiography showed a wide inner diameter of the aortic sinus. Abdominal ultrasonography was normal. In addition, no submucosal cleft, high arched/vaulted palate, hyperlaxity/hypermobile joint or dislocation was noticed.

### Molecular genetic studies

After obtaining informed consent, we isolated DNA from peripheral blood samples obtained from the patient and parents using the Gentra Puregene Blood Kit (Qiagen, Hilden, Germany). A whole exome library was captured by a SureSelect Human All Exon Kit (Agilent Technologies, Santa Clara, CA, USA) in accordance with the manufacturer’s instructions. Target regions were sequenced and aligned to the GRCh37/hg19 human reference sequence. Variants were annotated and filtered by TGex (https://fa.shanyint.com). Variants were classified following the American College of Medical Genetics and Genomics and the Association for Molecular Pathology (ACMG/AMP) standards and guidelines [3]. A homozygous nonsense variant c.100G > T / p.Gly34* in *SLC10A7* (NM_032128.4) was identified as putative pathogenic variant. Sanger sequencing confirmed the variant was inherited from both parents.

### Surgical treatments

The patient had her first surgery at 3.5 years old. During surgery, the spine was attached with single-side rods by interconnectors and fixed above and below the curve by pedicle screws at the vertebral body of T9, T10, L3, and L4 (Fig. 1L). She underwent lengthening twice at 4.5 and 5.5 years old respectively, and each time she was lengthened by 0.5 cm. From then, she had an X-ray every year to monitor the growth of her spine and progression of her scoliosis. At the age of 10.2, scoliosis progressed severely where the proximal end of single-side rods located. Therefore, surgery was conducted to change the single-side rods to dual rods to which pedicle screws were fixed at T1, T2, T3, T5, T8, T11, T12, L2, and L3 at the left side, and at T1, T2, T4, T5, T10, T12, L2, and L3 at the right side (Fig. 1M). She received lengthening surgeries twice again at the ages of 11.2 and 12.2, lengthened by 0.8 cm and 0.6 cm respectively. Notably, the surgeons had difficulty during every lengthening session and the effect was limited. The last surgery of final definite in situ fusion was performed at the age of 14.2 (Fig. 1N). At the last follow-up, she had a Cobb angle of 16.3° and no implant-related complications.

## Discussion

**Fig. 2:**
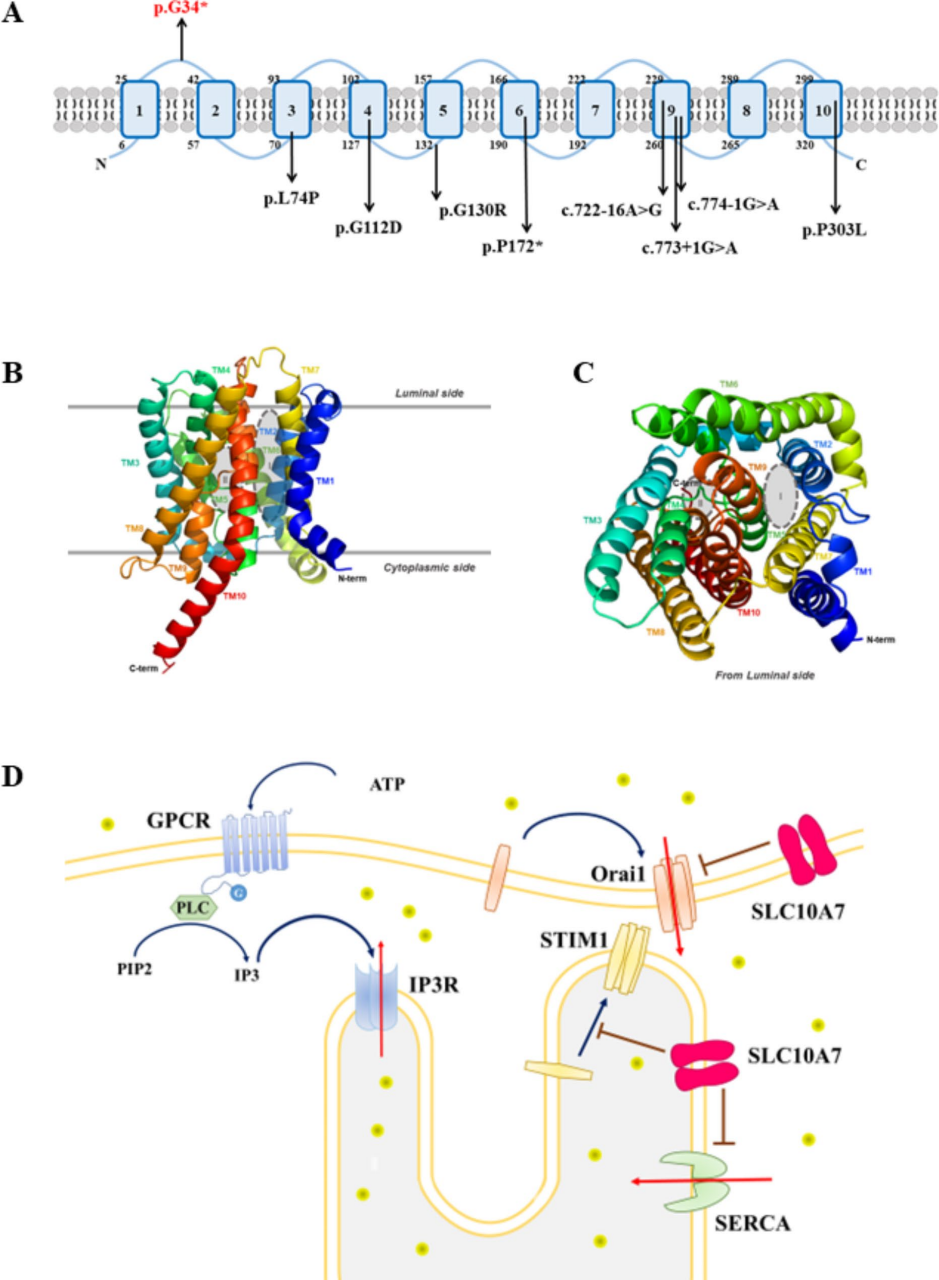
Schematic diagram of SLC10A7 protein and function. (**A**) Distribution of 12 reported variants. Nonsense variant of our case was labeled in red. **(B,C)** Predicted 3D structure of SLC10A7 protein. It was predicted using AphlaFold2 (https://www.alphafold.ebi.ac.uk/), and depicted using PyMOL (https://www.pymol.org). **(B)** Lateral side of protein. **(C)** Top side of protein depicted from luminal side. **(D)** SLC10A7 as a negative regulator of Ca^2+^ hemostasis by inhibiting Orai1 and SERCA. After GPCR activation, PLC hydrolyses PIP2 to IP3. The latter binds to IP3R and leads to depletion of ER Ca^2+^ store. ER Ca^2+^ sensor STIM1 oligomerizes and recruits Orai1 to ER-PM junction and stimulates Orai1-operated Ca^2+^ influx. Then, ER Ca^2+^ store is refilled by SERCA pumping cytoplasmic Ca^2+^ into ER. SLC10A7 could inhibit the STIM1, Orai1 and SERCA to negatively regulate Ca^2+^ uptake, thus SSASKS-causing SLC10A7 mutations increase Ca^2+^ influx. Abbreviations, TM, trans-membrane; N-term, N termination of protein; C-term, C termination of protein; GPCR, G protein-coupled receptors; PLC, phospholipase C; PIP2, phosphatidylinositol (4, 5) bisphosphate (PI (4, 5) P2); IP3, inositol triphosphate; IP3R, IP3 receptor; SERCA2, sarcoplasmic/endoplasmic reticulum calcium ATPase 2

After sequencing, we identified a novel homozygous nonsense variant c.100G > T / p.Gly34* in *SLC10A7* (NM_032128.4), which was inherited from her parents. This variant has not been reported in the dbSNP, 1000 genome, ESP, ExAC, or gnomAD databases, which indicates that it is rare in the normal population, neither not been previously reported in patients with SSASKS (Fig. 2A, Supplementary Table 1). It is located at the end of exon 1 of *SLC10A7*, and leads to the premature SLC10A7 protein. Therefore, we classified this nonsense variant as a pathogenic variant according to the ACMG/AMP guidelines.

Our patient had a relatively severe and broad clinical phenotype compared with previously reported cases (Supplementary Table 1). Similar to other cases of SSASKS, she had a short stature, type III hypocalcified amelogenesis imperfecta [2], and skeletal dysplasia characterized by short long bones, abnormal hands, progressive scoliosis, hyperlordosis, and kyphosis. Unlike other patients, both of her vision and hearing were compromised, and a wide inner diameter of the aortic sinus and patent foramen ovale were also found. Although no neurodevelopmental delay was observed to date, long-term follow-up and close attention are required. The patient received seven surgeries, which comprised rod implantation twice, lengthening four times, and final fusion once. Timely surgeries and regular check-ups effectively controlled the progression of scoliosis and possible kyphosis, and avoided implant-related complications [4]. Although there was limited growth potential of the spine in our patient with SSASKS, four lengthening surgeries helped to increase the patient’s height by 2.4 cm.

SSASKS is an extremely rare autosomal recessive disorder, which mainly affects the skeletal system. Because of characteristic abnormal N-glycosylation of the protein or lipid and overlapping skeletal defects, SSASKS was identified as a subtype of congenital disorders of glycosylation [5]. Part of patients with SSASKS have developmental delay in the intrauterine and postnatal periods which characterized by a short stature. Amelogenesis imperfecta, characterized by hypomineralized and discolored enamel [6], is a distinguishing feature of patients with SSAKS. Skeletal dysplasia can also affect the spine, limbs, hands and feet, and related joints. Irregular vertebral formation can lead to progressive scoliosis, hyperlordosis, or kyphoscoliosis. The limbs in these patients have short long bones, a distinct appearance of “Swedish key”, and abnormal epiphyses. The affected joints are hypermobile, and patients have knee dislocations, coxa valga, genua valga, and pes planus. Radiographs show advanced carpal and/or tarsal ossification of patients with SSASKS. Variable features include intellectual developmental impairment, facial dysmorphism, and hearing and visual impairment. Some of these patients can have a flat face, micrognathia, retrognathia, blue sclerae, hypertelorism, prominent eyes, and ptosis (Supplementary Table 1). In 2018, the first group of patients with SSASKS was identified, and this condition was related to *SLC10A7* mutation and secondary glucosaminoglycan (GAG) biosynthesis defects [7]. These defects include overrepresented high-mannose-type N-glycans, which is only composed of N-acetylglucosamine and mannose residues, and decreased sialylated complex-type (mature) N-glycans. To date, there have only been 12 cases of SSASKS reported worldwide (Fig. 2A, Supplementary Table 1) [1, 8].

SLC10A7 transporter is located at the compartments of the secretory pathway, including the endoplasmic reticulum (ER), Golgi, and plasma membrane (PM) in homodimers [9]. *SLC10A7* was first sequenced by Zou et al. who named it *C4orf13* [10]. *SLC10A7* has 10 possible TM domains and a putative O-linked glycosylation site (Fig. 2B, C), and both N and C termination of SLC10A7 are toward the cytosolic side [10]. Similar to its homolog from *Yersinia frederiksenii* (ASBT_Yf_) [11, 12] and *Neisseria meningitidis* (ASBT_NM_) [13], SLC10A7 contains a typical sodium/bile acid cotransporter family (SBF) domain as other members of the SLC10 family, with a core ion-binding site (Fig. 2B, C, gray area II) and an organic compound-transporting site (Fig. 2B, C, gray area I, between TM4 and TM9) [14]. However, SLC10A7 is not conserved with sequences of characteristic Na^+^ binding sites of bacterial ASBT_Yf_, human SLC10A1, and SLC10A2, and SLC10A7 does not transport bile acid or Na^+^ [14]. As an orphan solute carrier, which ion and organic compounds SLC10A7 transports are unknown.

SLC10A7 is ubiquitously expressed [15], and it is highly expressed in cartilages differentiating into long bones and growth plates [7]. In addition, in situ hybridization labeling in mouse fetuses and RT-PCR have shown precise spatiotemporal expression of *Slc10a7* in the teeth, vertebrae, and long bones. From embryonic day 14.5 (E14.5), *Slc10a7* expression is present at the epithelial compartment of the tooth cap stage. At E16.5, *Slc10a7* expression is found in the inner dental epithelium and in the epithelial loop of bell stage teeth. At E18.5, *Slc10a7* expression is present in the inner dental epithelium of the incisors and in ameloblasts and odontoblasts of the molars. During the process of ossification, vertebrae at E16.5 and E18.5, and the humerus and femur at E16.5 show *Slc10a7* expression [8, 16]. These findings suggest an important role of SLC10A7 in mineralization and ossification of the teeth, vertebrae, and long bones.

SLC10A7 transports glycoproteins from the post-Golgi compartment to the cell PM, and mediates bone mineralization [1]. Patients with SSASKS have a shift in the N-glycoprotein pattern, including increased high-mannose glycans and glycans lacking GlcNAc, and decreased sialylated glycans [7, 17]. SiaNAl labeling and pulse-chase labeling in fibroblasts of patients with SSASKS showed abnormal accumulation of sialylated glycoconjugates compared with healthy controls [17]. Zebrafish and a mouse model recapitulated the human phenotype of SSASKS. High-dose morpholino-induced *SLC10A7*-deficient zebrafish show a considerable reduction in bone mineralization at a nearly undetectable level [1]. A previous study showed that the *Slc10a7*^−/−^ mouse displayed a rounded skull, short long bones, disorganized growth plate, and hypomineralized AI [7]. Safranin O and Masson’s trichrome staining of the distal femur epiphysis showed disorder in the growth plates. The proliferative zone was thinner in the *Slc10a7*^−/−^ mouse owing to tightly packed chondrocytes. The pre-hypertrophic/hypertrophic zone was the most affected, and the hypertrophic layer was only limited to irregularly aligned two- to three-cell tiers. At the same time, stronger blue staining of collagen fibers was observed in the growth plates of *Slc10a7*^*−/−*^ mice than that in the wild type. This finding suggests an altered composition of the extracellular matrix, possibly due to a decreased proteoglycan/collagen ratio, leading to disorganization of the growth plate and a delay in bone growth. Patients with SSASKS and the *Slc10a7*^−/−^ mouse have a reduced proportion of heparan sulfates in total GAG, which suggests a defect in GAG synthesis because of SLC10A7 deficiency [7]. Therefore, SLC10A7 deficiency leads to abnormal Golgi glycosylation and a defect in GAG synthesis, including intracellular mis-localization of glycoproteins, a defect in post-Golgi transport of glycoproteins to the cell PM, and a reduced proportion of heparan sulfates.

SLC10A7 also negatively regulates cellular calcium hemostasis, and the pathogenic *SLC10A7* variant can reduce cellular calcium influx [8, 18]. Previous studies have shown that CaRch1 and ScRch1, which are two homologs of SLC10A7 in *Candida albicans* [19, 20] and *Saccharomyces cerevisiae* [21], function as regulators of cytosolic Ca^2+^ homeostasis and are regulated by the calcium/calcineurin signaling pathway. Fibroblasts in SLC10A7-deficienct patients show higher extracellular Ca^2+^ levels than those in healthy controls [7]. Two disease-causing missense variants (P303L and L74P) transfecting HEK293 cells also show higher Ca^2+^ influx levels than wild-type controls [18]. SLC10A7 negatively regulates Ca^2+^ influx by interacting with store-operated calcium entry (SOCE) and/or sarcoplasmic/ER calcium ATPase 2 (SERCA2) [9]. Extracellular ATP initiates G protein-coupled receptor (GPCR) to activate phospholipase C (PLC), which catalyzes phosphatidylinositol 4,5-diphosphate2 (PIP_2_) into inositol triphosphate (IP_3_). IP_3_ receptor (IP_3_R), which is located at the ER, interacts with IP_3_ and transports ER luminal Ca^2+^ into the cytosol. Stromal interaction molecule 1 (STIM1) senses a reduction of Ca^2+^ in the ER and recruits Orai1 from the PM to the PM-ER junction and activates Orai1-mediated Ca^2+^ influx. Finally, SERCA2 at the ER pumps Ca^2+^ from the cytosol to the ER lumen and restores Ca^2+^ levels in the ER (Fig. 2D). SLC10A7 can inhibit STIM1, Orai1, and SERCA2 to negatively regulate intracellular calcium signaling, and this might be involved in Golgi glycosylation and GAG synthesis mentioned above.

## Conclusion

We report the first case of SSASKS in a Chinese individual with a novel *SLC10A7* variant. SSASKS is an autosomal recessive skeletal disorder. SSASKS is characterized by multi-region skeletal dysplasia. Our patient had a homozygous *SLC10A7* pathogenic nonsense variant and showed a relatively severe clinical phenotype, which resembled previously reported cases. Timely surgery and regular check-ups could preserve the growth potential of the spine, while limiting the progression of spinal curvature. The exact intracellular localization of SLC10A7 and the ions and organic compounds it transports still need to be clarified. However, a deficiency of SLC10A7, which leads to a defect in glycosylation and calcium homeostasis, highlights the important role of SLC10A7 in skeletal ossification.

### Electronic supplementary material

Below is the link to the electronic supplementary material.

Supplementary Material 1

Supplementary Material 2

## Data Availability

The raw sequence data reported in this paper have been deposited in the Genome Sequence Archive (Genomics, Proteomics & Bioinformatics 2021) in National Genomics Data Center (Nucleic Acids Res 2022), China National Center for Bioinformation / Beijing Institute of Genomics, Chinese Academy of Sciences (GSA-Human: HRA005653) that are publicly accessible at https://ngdc.cncb.ac.cn/gsa-human.

## Abbreviations

ACMG/AMP: American College of Medical Genetics and Genomics and the Association for Molecular Pathology
AI: Amelogenesis imperfecta
E: Embryonic day
ER: Endoplasmic reticulum
GAG: Glucosaminoglycan
IP_3_: Inositol triphosphate
IP_3_R: Inositol triphosphate receptor
PIP_2_: Phosphatidylinositol 4,5-diphosphate2
GPCR: G protein-coupled receptor
PLC: Phospholipase C
PM: Plasma membrane
SBF: Sodium/bile acid cotransporter family
SERCA2: Sarcoplasmic/ER calcium ATPase 2
SLC10A7: Solute carrier family 10 member 7
SOCE: Store-operated calcium entry
SSASKS: Short stature, amelogenesis imperfecta, and skeletal dysplasia
STIM1: Stromal interaction molecule 1
TM: Transmembrane

## References

[1] Ashikov A, Bakar NA, Wen X-Y, Niemeijer M, Osorio GRP, Brand-Arzamendi K (2018). Integrating glycomics and genomics uncovers SLC10A7 as essential factor for bone mineralization by regulating post-golgi protein transport and glycosylation. Hum Mol Genet.

[2] Sabandal MMI, Schäfer E (2016). Amelogenesis Imperfecta: review of diagnostic findings and treatment concepts. Odontology.

[3] Richards S, Aziz N, Bale S, Bick D, Das S, Gastier-Foster J (2015). Standards and guidelines for the interpretation of sequence variants: a joint consensus recommendation of the American College of Medical Genetics and Genomics and the Association for Molecular Pathology. Genet Medicine: Official J Am Coll Med Genet.

[4] Sebaaly A, Daher M, Salameh B, Ghoul A, George S, Roukoz S (2022). Congenital scoliosis: a narrative review and proposal of a treatment algorithm. EFORT open Reviews.

[5] Ng BG, Freeze HH (2018). Perspectives on glycosylation and its congenital disorders. Trends Genet.

[6] Crawford PJ, Aldred M, Bloch-Zupan A (2007). Amelogenesis Imperfecta. Orphanet J Rare Dis.

[7] Dubail J, Huber C, Chantepie S, Sonntag S, Tüysüz B, Mihci E (2018). SLC10A7 mutations cause a skeletal dysplasia with amelogenesis imperfecta mediated by GAG biosynthesis defects. Nat Commun.

[8] Laugel-Haushalter V, Bär S, Schaefer E, Stoetzel C, Geoffroy V, Alembik Y (2019). A New SLC10A7 homozygous missense mutation responsible for a milder phenotype of skeletal dysplasia with Amelogenesis Imperfecta. Front Genet.

[9] Karakus E, Wannowius M, Müller SF, Leiting S, Leidolf R, Noppes S (2020). The orphan solute carrier SLC10A7 is a novel negative regulator of intracellular calcium signaling. Sci Rep.

[10] Zou X, Wang D, Qiu G, Ji C, Jin F, Wu M (2005). Molecular cloning and characterization of a novel human C4orf13 gene, tentatively a member of the sodium bile acid cotransporter family. Biochem Genet.

[11] Zhou X, Levin EJ, Pan Y, McCoy JG, Sharma R, Kloss B (2014). Structural basis of the alternating-access mechanism in a bile acid transporter. Nature.

[12] Wang X, Lyu Y, Ji Y, Sun Z, Zhou X (2021). Substrate binding in the bile acid transporter ASBT(yf) from Yersinia frederiksenii. Acta Crystallogr Sect D Struct Biology.

[13] Hu NJ, Iwata S, Cameron AD, Drew D (2011). Crystal structure of a bacterial homologue of the bile acid sodium symporter ASBT. Nature.

[14] Durin Z, Dubail J, Layotte A, Legrand D, Cormier-Daire V, Foulquier F (2022). SLC10A7, an orphan member of the SLC10 family involved in congenital disorders of glycosylation. Hum Genet.

[15] Godoy JR, Fernandes C, Döring B, Beuerlein K, Petzinger E, Geyer J (2007). Molecular and phylogenetic characterization of a novel putative membrane transporter (SLC10A7), conserved in vertebrates and bacteria. Eur J Cell Biol.

[16] Laugel-Haushalter V, Langer A, Marrie J, Fraulob V, Schuhbaur B, Koch-Phillips M (2012). From the transcription of genes involved in ectodermal dysplasias to the understanding of associated dental anomalies. Mol Syndromol.

[17] Ashikov A, Abu Bakar N, Wen XY, Niemeijer M, Rodrigues Pinto Osorio G, Brand-Arzamendi K (2018). Integrating glycomics and genomics uncovers SLC10A7 as essential factor for bone mineralization by regulating post-golgi protein transport and glycosylation. Hum Mol Genet.

[18] Wannowius M (2021). Functional analysis of Rare Genetic variants in the negative Regulator of Intracellular Calcium Signaling RCAS/SLC10A7. Front Mol Biosci.

[19] Jiang L, Alber J, Wang J, Du W, Yang X, Li X (2012). The Candida albicans plasma membrane protein Rch1p, a member of the vertebrate SLC10 carrier family, is a novel regulator of cytosolic Ca2 + homoeostasis. Biochem J.

[20] Alber J, Jiang L, Geyer J (2013). CaRch1p does not functionally interact with the high-affinity ca(2+) influx system (HACS) of Candida albicans. Yeast (Chichester England).

[21] Zhao Y, Yan H, Happeck R, Peiter-Volk T, Xu H, Zhang Y (2016). The plasma membrane protein Rch1 is a negative regulator of cytosolic calcium homeostasis and positively regulated by the calcium/calcineurin signaling pathway in budding yeast. Eur J Cell Biol.

