# Supplementary figures and images for: Identification of novel homozygous nonsense SLC10A7 variant causing short stature, amelogenesis imperfecta, and skeletal dysplasia with scoliosis and surgical management of spine
Source: Orphanet J Rare Dis. 2023 Nov 30;18:371. doi: 10.1186/s13023-023-02975-0 (PMC10691085; doi:10.1186/s13023-023-02975-0)

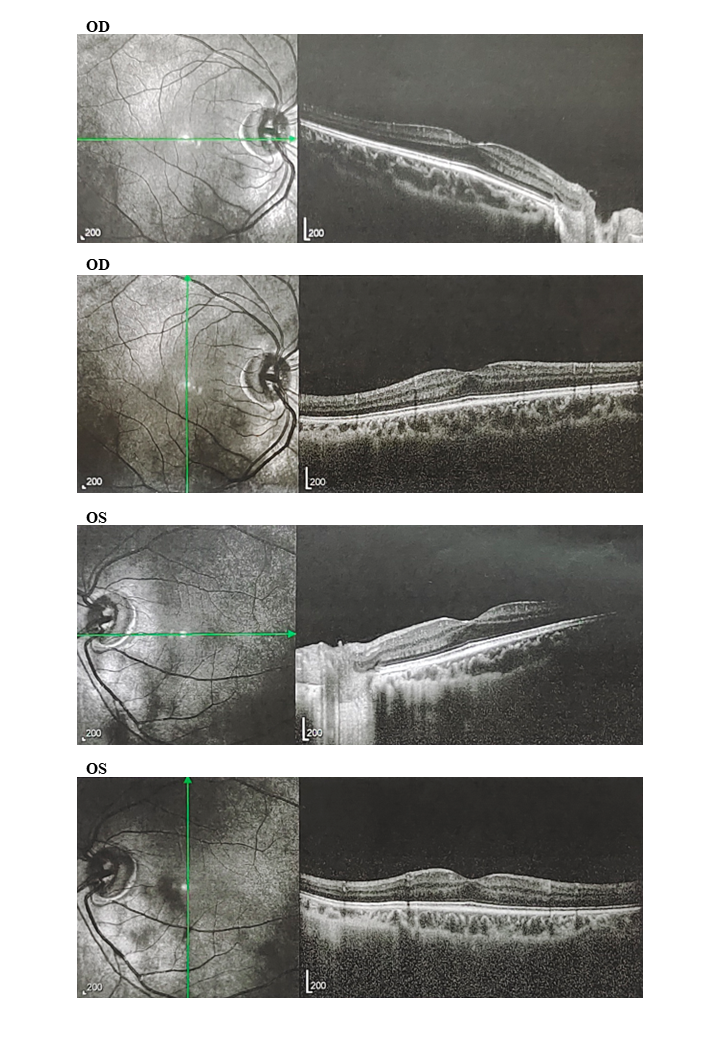

Supplement: Supplementary file 2 — Supplementary Material 2 [file 13023_2023_2975_MOESM2_ESM.tif]
